# Baclofen Modulates Neural Intrinsic Functional Connectivity in Treatment‐Seeking Individuals With Alcohol Use Disorder

**DOI:** 10.1111/acer.70332

**Published:** 2026-06-05

**Authors:** Warren B. Logge, Tristan P. Hurzeler, Paul S. Haber, Laurence Mealing, Andrew J. Baillie, Kirsten C. Morley

**Affiliations:** ^1^ Edith Collins Centre for Translational Research in Alcohol, Drugs and Toxicology Royal Prince Alfred Hospital, Sydney Local Health District Sydney New South Wales Australia; ^2^ Specialty of Addiction Medicine, Central Clinical School, Faculty of Medicine and Health University of Sydney Sydney New South Wales Australia; ^3^ Drug Health Services Sydney Local Health District Sydney New South Wales Australia; ^4^ Faculty of Health Sciences University of Sydney Sydney New South Wales Australia

**Keywords:** alcohol use disorder, baclofen, functional connectivity, functional magnetic resonance imaging, randomized controlled trial

## Abstract

**Background:**

Chronic alcohol use alters brain networks related to reward and stress regulation, contributing to alcohol use disorder (AUD). Baclofen, a GABA_B_ receptor agonist, reduces cue‐elicited neural responses and alcohol consumption, but its effects on intrinsic functional connectivity remain unclear. This study investigates the dose‐specific effects of baclofen on intrinsic resting‐state functional connectivity in treatment‐seeking individuals with AUD.

**Methods:**

In this double‐blind, placebo‐controlled, randomized clinical trial, 29 participants with AUD were randomized to receive placebo (*n* = 10), low‐dose baclofen (30 mg/day, *n* = 10), or high‐dose baclofen (75 mg/day, *n* = 9) for 12 weeks. Resting‐state fMRI data were collected at rest during week 2. A data‐driven parcellation approach assessed connectivity patterns across the brain and associations with percentage of heavy drinking days and percentage of days abstinent post scan were examined in an exploratory analysis.

**Results:**

Compared to placebo, high‐dose baclofen was associated with significant differences in intrinsic connectivity in brain regions linked with reward, stress, attentional, and salience networks. Higher connectivity was observed between the somatomotor network and regions involved in stress regulation (e.g., hypothalamus) and reward processing, while lower connectivity was observed within salience and attentional networks. These changes were not observed in the low‐dose or placebo groups. Although nonsignificant, associations between MES and clinical outcomes demonstrated moderate, directionally consistent effects for abstinence‐related outcomes.

**Conclusions:**

High‐dose baclofen may modulate intrinsic brain connectivity in key networks implicated in AUD, including systems involved in attention and stress regulation. Although statistically significant relationships between functional connectivity and clinical outcomes were not identified, trends suggest that connectivity differences between high‐dose baclofen and placebo may be relevant to treatment response. These neurobiological findings provide additional support for baclofen as a dose‐dependent pharmacotherapy for AUD and highlight the need for larger samples to clarify the relationship between intrinsic connectivity and clinical outcomes.

**Trial Registration:**

ClinicalTrials.gov, NCT01711125, https://clinicaltrials.gov/ct2/show/NCT01711125 letter of completion

## Graphical abstract

In a randomized, double‐blind study of individuals with alcohol use disorder, high‐dose baclofen (75 mg/day) compared with placebo was associated with differences in resting‐state functional connectivity, with higher connectivity in reward and stress‐related regions and lower connectivity in attentional and salience networks.

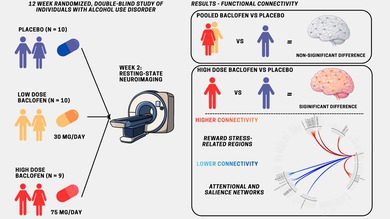

## 1 Introduction

Alcohol use disorder (AUD) is a common disorder marked by recurrent relapses into heavy drinking (Haber et al. 2021). Baclofen, a selective agonist of the γ‐aminobutyric acid (GABA)_B_ receptor, has gained attention as a potential treatment for alcohol use disorder, with a notable increase in its application (Logge et al. 2022). Baclofen can decrease alcohol cravings and improve drinking outcomes among individuals with AUD (Addolorato et al. 2002). However, findings from several large clinical trials have been inconsistent in demonstrating the impact of baclofen on drinking behavior (Addolorato et al. 2002; Beraha et al. 2016; Garbutt et al. 2010; Morley et al. 2018; Müller et al. 2015; Reynaud et al. 2017). These mixed results underscore the need for further research to clarify the mechanisms that underlie the effects of baclofen and help inform the optimal clinical use of baclofen in AUD treatment.

Meta‐analyses have yielded varied results on the efficacy of baclofen. Some analyses link baclofen to higher abstinence rates compared to placebo in heavy drinking AUD populations (Pierce et al. 2018). The largest meta‐analysis to date reported only a modest advantage for baclofen over placebo, particularly in uncomplicated AUD cases (Bschor et al. 2018). Importantly, a recent Cochrane systematic review and meta‐analysis of 17 randomized controlled trials (1818 participants) found that baclofen likely reduces the risk of relapse to any drinking and increases the percentage of abstinent days compared with placebo, particularly among detoxified individuals and at lower doses (Agabio et al. 2023). However, a more recent meta‐analysis in AUD patients with liver disease found no significant difference in abstinence rates between baclofen and placebo, highlighting the need for additional research in this subgroup (Duan et al. 2023). Dose–response effects have also been observed, with efficacy primarily at lower doses (< 60 mg/day) for promoting abstinence (Pierce et al. 2018), though there is considerable variability in the dosage required to reduce alcohol cravings across individuals (De Beaurepaire and Jaury 2024). The expert Cagliari Consensus statement suggests that while baclofen's superiority over placebo has not been firmly established, further exploration of dose–response dynamics is essential (Agabio et al. 2018), particularly given the reported side effect profile regarding potential for sedation (Rolland et al. 2015).

Baclofen shows potential in dampening conditioned drug‐related responses and targeting cue‐triggered vulnerabilities linked to relapse in substance use disorders as shown through functional magnetic imaging approaches that can highlight the mechanisms underpinning pharmacotherapeutic action. There is considerable evidence from neuropharmacological studies that baclofen blocks conditioned drug responses in AUD, which may extend to attenuating drug cue reactivity in humans (e.g., [Beck et al. 2018; Beraha et al. 2016; Farokhnia et al. 2018; Farokhnia et al. 2017; Logge et al. 2021]). This attenuation of drug reward‐related neural responses has also been observed in other substance use disorders, including treatment‐seeking abstinent individuals with cocaine use disorder treated with baclofen (60 mg/day) compared to placebo (Young et al. 2014). Additionally, Pelz et al. (2023) found that individually titrated high‐dose baclofen (30‐270 mg) significantly decreased insula cortex activity in alcohol‐dependent patients performing a slot machine task, suggesting baclofen may help attenuate gain anticipation and thus exerting a positive effect on general reward processing in this population. These findings underscore a potentially common mechanism by which baclofen dampens neural responsiveness to cues, particularly within regions implicated in craving and relapse such as anterior cingulate cortex (ACC) and bilateral dorsolateral prefrontal cortex (dlPFC), while reducing responses in the ventral striatum (VS) and regions within the orbitofrontal and insular cortices.

Another informative fMRI neuroimaging approach that can evaluate neural treatment responses is functional intrinsic connectivity analysis, which examines spontaneous BOLD fluctuations that reflect the inherent neuronal activity in the brain while individuals are awake (Biswal 2012). Intrinsic functional connectivity is emerging as a promising biomarker for assessing treatment responses in alcohol and substance use disorders, offering potential for gauging the effectiveness of new treatments in early‐phase research (Biswal 2012; Tolomeo and Yu 2022; Wilcox et al. 2019). Functional connectivity changes have been observed during specific tasks after baclofen treatment, highlighting its effects on neural activation. Beck et al. (2018) reported that during baclofen treatment (30–270 mg/day), alcohol cue‐modulated functional connectivity was altered. Specifically, decreased connectivity was observed between the ventral tegmental area (VTA) and the left medial prefrontal cortex (MPFC) and anterior cingulate cortex (ACC), alongside increased connectivity between the VTA and the right MPFC. The authors concluded the reduced coupling may reflect diminished salience (appetitive and aversive) of alcohol‐related stimuli, as the ACC and MPFC—that comprise part of an “attention network”—are sensitive to drug cues. Baclofen may thus lower attention allocation to such cues without affecting connectivity independent of cue modulation. A perfusion MRI study of smokers found that baclofen (80 mg/day) may reduce cue‐induced craving and relapse risk by enhancing baseline resting‐state activity in the dlPFC, an executive control region, which in turn diminishes neural responses in craving‐related areas during cue exposure, such as the insula and ventromedial prefrontal cortex (vmPFC) (Ketcherside et al. 2020). These findings support the potential for baclofen to assist individuals sensitive to drug cue‐elicited urges to reduce relapse through altering brain activity patterns at rest, and thus a potentially promising intervention in treating substance use disorders where cue reactivity is a significant factor. However, no studies to date have specifically investigated changes in intrinsic functional connectivity in AUD related to baclofen treatment.

This study therefore aimed to assess whether heavy drinkers treated with baclofen, compared to placebo, exhibit distinct patterns of intrinsic functional connectivity at rest. This is the first study to explore the effects of baclofen on intrinsic functional connectivity in AUD. Few studies have applied functional connectivity methods to examine the effects of baclofen in AUD, and most of these focus on cue‐induced rather than resting‐state activity. To identify potential treatment‐related connectivity differences, we employed a data‐driven whole‐brain parcellation approach. We hypothesized that baclofen would decrease activation in brain regions involved in reward processing, particularly within mesocorticolimbic circuits.

## 2 Materials and Methods

### 2.1 Study Design

Participants were randomized to receive placebo, or low dose (30 mg/day) or high dose (75 mg/day) baclofen for 12 weeks. The study was conducted over a 36‐month period at three sites (Royal Prince Alfred Hospital, North Shore Hospital and Westmead Hospital) in Australia between 2013 and 2016. At approximately the second week of the trial (*M* = 17 days), participants underwent an fMRI resting state scan. The study was approved by the Human Ethics Review Committee of the Sydney Local Health District (X11‐0154). The study involved off‐label use of a registered medication in Australia and approval was given under the Clinical Trial Notification (CTN) scheme of the Therapeutics Goods Administration (TGA) (2013/0060). All participants included in this MRI substudy provided written informed consent after commencement of randomization for the main trial.

### 2.2 Participants

Thirty‐four participants were recruited as part of a larger trial investigating the efficacy of baclofen treatment in alcohol dependence (Morley et al. 2013), with the primary outcomes (Morley et al. 2018) and fMRI cue reactivity outcomes previously reported (Logge et al. 2021; Morley et al. 2021). Participation in the neuroimaging substudy was discussed with participants after eligibility for the main study was confirmed; participation was voluntary and subject to standard MRI eligibility criteria. Inclusion criteria included: (i) Alcohol dependence according to the ICD‐10 criteria; (ii) Age 18–75; (iii) Adequate cognition and English language skills to give valid consent and complete research interviews; (iv) Willingness to give written informed consent; (v) Abstinence from alcohol for between 3 and 21 days leading up to randomization; (vi) Resolution of any clinically evident alcohol withdrawal (CIWA‐Ar; (Sullivan et al. 1989)); (vii) Less than 48 h after ceasing any diazepam required for withdrawal management. For stratification, alcoholic liver disease (ALD) was defined as the presence of symptoms and/or signs referable to liver disease or its complications with or without cirrhosis, in which alcohol use was considered to play a major etiological role. Alcohol use had to exceed an average of 60 g/day in women and 80 g/day in men for > 10 years. If other co‐factors such as chronic hepatitis C were present, a significant contribution of alcohol to liver disease was considered present if a period of supervised abstinence (e.g., in hospital) led to a ≥ 50% improvement in liver enzymes.

Exclusion criteria were: (i) Active major mental disorder associated with psychosis or significant suicide risk, (ii) Pregnancy or lactation, (iii) Concurrent use of any psychotropic medication other than antidepressants (provided these were taken at stable doses for at least 2 months); (iv) Unstable substance use; (v) Clinical evidence of persisting hepatic encephalopathy (drowsiness, sleep inversion, or asterixis); (vi) Pending incarceration; (vii) Lack of stable housing, (viii) Peptic ulcer; and (ix) Unstable diabetes mellitus. Additional MRI‐specific exclusion criteria included standard safety contraindications such as claustrophobia, extreme obesity incompatible with scanner limits, history of brain surgery, occupational exposure to metal fragments (e.g., machinists, welders, metal workers), or the presence of metallic or electronic implants (e.g., pacemakers, aneurysm clips, metal implants, hearing aids, prosthetic devices, insulin pumps, or metallic fragments in the body).

Participants completing imaging were primarily alcohol‐dependent participants without ALD to reduce potential confounding effects of severe liver disease symptoms on brain morphology (e.g., hepatic encephalitis), and those ALD participants that were scanned (*n* = 6, 2 participants per dose group) had relatively low liver disease severity as determined by the modified Model End‐stage Liver Disease (Forman and Lucey 2001) score (*M* = 8.71, SD = 3.32), a measure utilizing objective parameters of disease severity. Three participants had a breath alcohol level (BrAC) exceeding 0.00 and their test session was concluded, and two participants presented significant cognitive dysfunction, and their results were not included in the main effects analyses (sample *N* = 29).

### 2.3 Procedure

Participants underwent a structured interview and medical consultation on day 0 assessing trial eligibility. For the main trial, participants were randomized 1:1:1 to baclofen to take a capsule of 10 or 25 mg for 84 days, with medication titrated upward for the first 4 days to full dose of three capsules per day and titrated downward during the last four trial days. The placebo capsules were identical in appearance and also followed the same titration schedule to maintain the double blind. Participants then completed baseline questionnaires.

Imaging sessions were scheduled at approximately week 2 on the trial and were conducted between 10 a.m. and 4 p.m. This timing allowed participants to reach and stabilize on their assigned baclofen dose following the initial titration period. Participants completed resting state scan approximately 120 min after administration of their first daily relevant medication capsule (10 mg or 25 mg baclofen, placebo).

### 2.4 Assessments

#### 2.4.1 Baseline (Week 0, Day 0)

Consumption in the preceding 30 days before enrolment was measured using the Timeline followback interview (TLFB) (Sobell and Sobell 1992) in number of Australian standard units (10 g ethanol) per drinking day (henceforth TLFB units). The Alcohol Dependence Scale (ADS) (Skinner and Allen 1982) measured participants' AUD severity, with higher scores indicating greater severity. Drinkers Inventory of Consequences (DrInC) Lifetime (Miller et al. 1995) indicated physical, emotional, and social consequences related to alcohol ever being experienced, with higher DrInC scores indicating more negative drinking consequences.

#### 2.4.2 Scanning (Week 2)

A 7‐day TLFB was first completed assessing participants' previous week of consumption.

### 2.5 MRI Data Acquisition

MRI data were acquired on a 3‐Tesla GE Discovery (GE Healthcare, Milwaukee, Wisconsin, United States) using a 32‐channel head coil. A T1‐weighted (1‐mm^3 voxel resolution) structural scan was acquired for each subject for screening and registration (TR: 7200 ms, TE 2.7 ms, 176 sagittal slices, 1 mm thick, no gap, 256 × 256 × 256 matrix). For BOLD acquisition, we acquired 203 echoplanar image (EPI) volumes comprising 39 axial slices in an ascending interleaved fashion with a voxel resolution of 1.88 × 1.88 × 2 mm (TR: 3000 ms, TE 30 ms, FA 90̊, FOV 240 mm, matrix 128 × 128, acceleration factor 2, slice gap: 1 mm). Participants' heads were fixated with foam pads to minimize head movement. For the fMRI resting‐state acquisition, participants were told to focus on the fixation cross displayed on the presentation screen and to leave their eyes open while thinking of nothing in particular. This resting‐state acquisition was acquired prior to the task‐based fMRI tasks (Logge et al. 2021; Morley et al. 2021) to avoid any carryover from stimulus‐elicited neural activity.

### 2.6 Image Processing

A detailed overview of imaging processing pipeline is provided in the Supporting Information, with a summary provided here. Anatomical and functional preprocessing was completed using FMRIPrep ([Esteban et al. 2020; Esteban et al. 2019], RRID:SCR_016216), with the T1‐weighted structural images corrected for intensity non‐uniformity, skull‐stripped, and then used for reconstruction of the brain surfaces. Volume‐based structural images were segmented into white matter, gray matter, and cerebrospinal fluid, and then spatially normalized into MNI space. Functional resting‐state MRI data per subject were preprocessed with susceptibility distortion correction using a B0‐nonuniformity map (i.e., fieldmap) using a deformation field to correct for susceptibility distortions estimated based on fMRIPrep's fieldmap‐less approach. The deformation field is that resulting from co‐registering the BOLD reference to the same‐subject T1w‐reference with its intensity inverted. Images underwent motion correction, co‐registration to structural data, normalization to MNI space, and projection to cortical surface. Functional time series were then resampled to FreeSurfer's (FreeSurfer 6.0.1, surfer.nmr.mgh.harvard.edu) fsaverage space. Resting‐state data were further post‐processed using eXtensible Connectivity Pipelines (XCP‐D) (Ciric et al. 2017; Satterthwaite et al. 2013). Volumes with framewise‐displacement (FD) greater than 0.4 were flagged as outliers and excluded. A total of 36 nuisance regressors were regressed out of the BOLD data using the “36P” strategy (Ciric et al. 2017; Satterthwaite et al. 2013). Residual time series were then band‐pass filtered (0.01–0.08 Hz) and spatially smoothed with a kernel size of 6 mm (FWHM). Fisher's r‐to‐z transformation was applied to the Pearson correlation coefficients per cell of the resultant matrices of the ROI‐to‐ROI connectivity matrix using the cortical–subcortical 256 Schaefer Supplemented with Subcortical Structures parcel atlas (https://github.com/PennLINC/AtlasPack/) for each subject outputted from the XCP‐D processing. Of the 256 parcels from the 17 functional networks including subcortical parcellations, 16 parcels contained > 50% missing data and were excluded from second‐level analyses (See Supporting Information for information), leaving a 243 × 243 symmetrical functional connectivity matrix per participant.

### 2.7 Statistical Analysis

Resting state data were analyzed using CONN toolbox (Whitfield‐Gabrieli and Nieto‐Castanon 2012) version 22a (Nieto‐Castanon and Whitfield‐Gabrieli 2021). The 243 × 243 ROI‐to‐ROI *z* score correlation matrices were entered into a second‐level GLM model with between‐subjects treatment (baclofen > pooled placebo [i.e., 1–1]) evaluating the effect of pooled baclofen versus placebo. Sex and antidepressant use were included as covariates of no interest. To align with our previous fMRI studies evaluating dose‐specific effects, we employed two separate second‐level GLM models with between‐subjects treatment for the separable doses compared to placebo, for low‐dose baclofen (low‐dose baclofen > placebo [i.e., 1–1]) and for high‐dose baclofen (high‐dose baclofen > placebo [i.e., 1–1]). A parametric multivariate statistics approach was conducted to target the contributions of the individual ROIs. An FDR‐corrected ROI‐level *p*‐value (MVPA omnibus test) was used with a threshold of P_FDR_ < 0.05 (Benjamini and Hochberg 1995) with a connection (height) threshold of *p* < 0.01 (uncorrected) to examine the individual connections between the ROIs.

In exploratory analysis, the relationship between functional connectivity and clinical outcomes was calculated by deriving mean edge strength (MES) values from significant connectivity patterns. For each participant, MES was calculated by averaging Fisher z‐transformed connectivity values across relevant ROI‐to‐ROI connections. This provided a summary measure of overall connectivity strength. Pearson correlation coefficients were then used to examine associations between MES (positive and negative) and post‐scan drinking outcomes, including percentage of heavy drinking days (HDD%), percentage of days abstinent, and time to relapse. *p*‐values were adjusted for multiple comparisons using the Benjamini–Hochberg false discovery rate (FDR) correction procedure. Given the potential instability of correlation estimates and the modest sample size, robustness of observed associations was assessed using nonparametric bootstrapping. Bias‐corrected and accelerated (BCa) bootstrap confidence intervals (CIs) were estimated using 1000 resamples. This allowed for the estimate of the sampling distribution, allowing for a more reliable inference by quantifying the stability and uncertainty of effect sizes.

## 3 Results

Table 1 summarizes the demographic and clinical characteristics of the sample and drinking outcomes. There were no significant differences between placebo and pooled baclofen groups (*p*'s > 0.206), or across the three treatment groups (placebo, low‐dose BAC, high‐dose BAC; *p*'s > 0.168) for age, gender, years of education, or unemployment. Similar levels of craving were seen at baseline represented by the PACS, number of years since alcohol‐related problems began, and levels of alcoholic liver disease, tobacco use, and antidepressant use for both the placebo versus pooled BAC comparison (*p*'s > 0.073) and across the three treatment groups (*p*'s > 0.175). When comparing placebo versus the pooled baclofen group, those randomized to placebo had marginally higher reported baseline drinks per drinking day (*p* = 0.049), but no differences when evaluating three groups (*X* ^2^ = 0.95, *p* = 0.623).

**TABLE 1:** Sample Characteristics with Tests of Group Differences.

	Placebo (*n* = 10)	BAC			Test for differences	
		30 mg/day (*n* = 10)	75 mg/day (*n* = 9)	Pooled (*n* = 19)	Placebo versus BAC	Three treatment levels
*Demographics*						
Age	53.20 ± 11.62	47.30 ± 5.83	52.33 ± 9.92	49.68 ± 8.21	0.206	0.168
Gender, % F		40	11	26	0.220	0.237
Education, years	14.60 ± 2.37	14.20 ± 2.82	14.22 ± 2.17	14.21 ± 2.46	0.710	0.716
Unemployed, %	20	40	33	36	0.375	0.706
*Clinical Characteristics*						
Drinks per drinking day^a^	12.71 ± 4.08	11.46 ± 7.32	8.20 ± 4.02	9.92 ± 6.06	0.**049**	0.623
Years since alcohol‐related problems began	21.3 13.39	16.50 ± 11.05	11.56 ± 8.17	14.16 ± 9.86	0.073	0.360
ADS score	13.6 ± 6.5	17.20 ± 5.81	16.00 ± 10.93	16.63 ± 8.39	0.475	0.311
PACS Craving	17.9 ± 5.38	15.90 ± 6.33	14.67 ± 5.02	15.32 ± 5.63	0.334	0.434
Alcoholic liver disease, %	30	20	22	21	0.620	0.861
Tobacco use, %	70	90	55	73	0.859	0.283
Antidepressant use, %	80	80	44	63	0.375	0.175

*Note:* Data represent *M* ± SDs, unless otherwise noted. Values in bold are statistically significant (*p* < 0.05).

Abbreviations: ADS, Alcohol Dependence Severity Scale; BAC, Baclofen; HDD, Heavy drinking days; PACS, Penn Alcohol Craving Scale.

^a^ During the 30 days preceding the first day of the study, based on the Time‐Line Follow‐Back method.

### 3.1 Intrinsic Functional Connectivity

**FIGURE 1:**
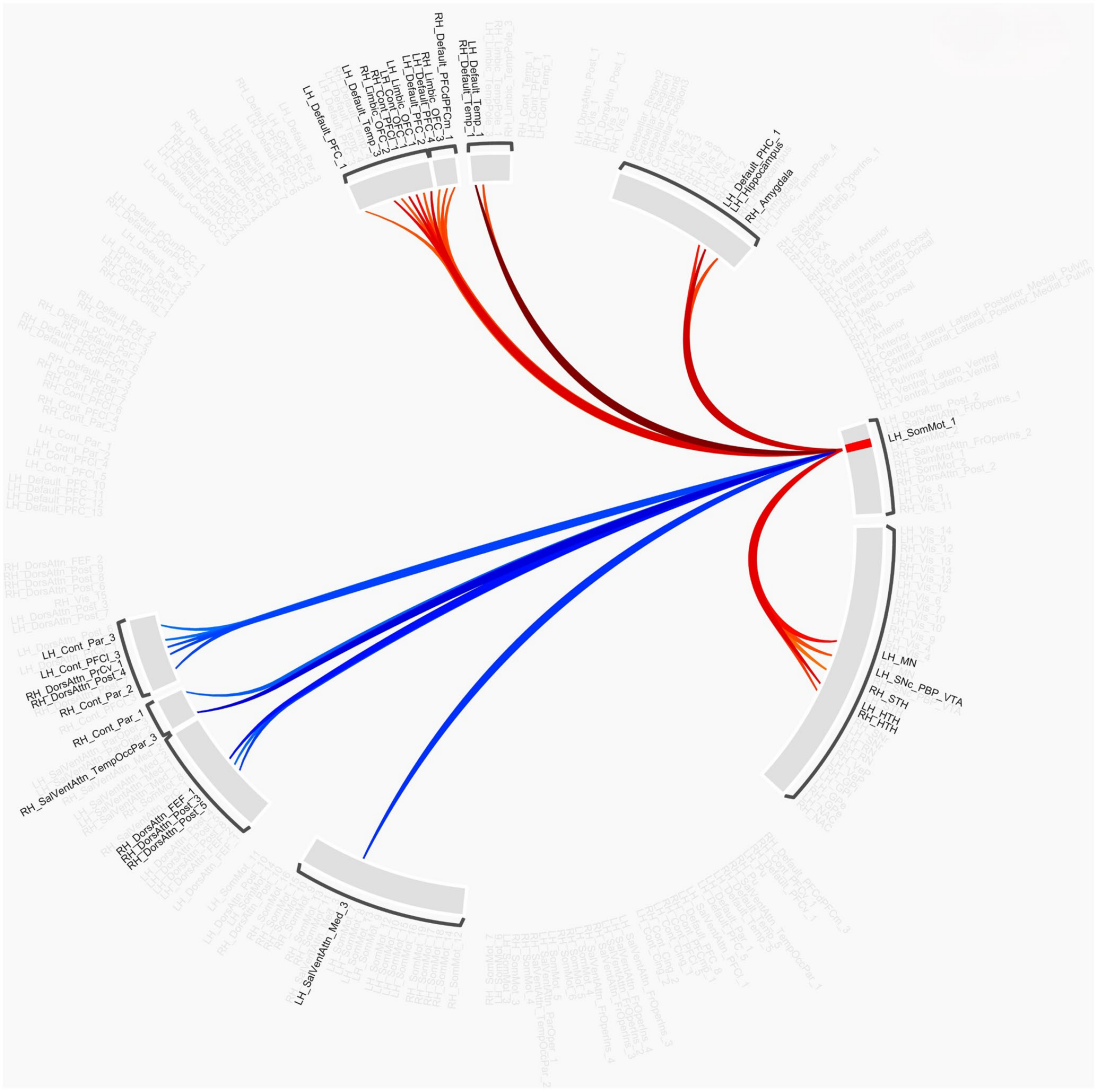
Significant ROI‐to‐ROI treatment connections comparing high‐dose baclofen‐ versus placebo‐treated patients. Nodes are displayed on the wheel for the Schaefer Supplemented with Subcortical Structures atlas with 256 parcel resolution. Colored connections represent significant connections between the left hemisphere somatomotor 1 node (highlighted red), with blue lines representing respective connections with lower functional connectivity, and red led lines representing connections with higher functional connectivity between nodes. These connections are significant at a nominal, uncorrected *p*‐value threshold of 0.01.

When comparing functional connectivity patterns between placebo and the pooled baclofen group, there was no evidence of significant differences seen at the treatment level, indicating the pooled baclofen group did not differ from placebo in intrinsic functional connectivity. When comparing separable doses versus placebo, there were no differences in functional connectivity patterns seen when evaluating the low‐dose BAC versus placebo. However, when comparing high‐dose baclofen with placebo, a significant treatment effect was observed, with both higher and lower functional connectivity patterns observed for BAC participants after treatment in one ROI seed region, somatomotor 1 (MVPA P_FDR_ < 0.0001) in the left hemisphere of the somatomotor network of the Schaefer 200 atlas parcellation. Higher connectivity was observed between target regions in networks and circuits that regulate emotional or stress reactivity, including the hypothalamus, subthalamic nucleus, and amygdala, and limbic OFC, the reward system including the ventral tegmental area and mammillary nucleus, and regions in the prefrontal cortex and temporal lobes in the default mode network. Lower connectivity was also observed in target regions within the dorsal attentional and salience ventral attentional networks. Figure 1 presents these connections, with connectivity values reported in Supporting Information.

Exploratory analyses (Table 2) indicated no statistically significant associations between MES (positive or negative) and percentage of heavy drinking days (HDD%) or percentage of days abstinent after correction for multiple comparisons (p_FDR_ > 0.05). However, moderate effect sizes were observed for percentage of days abstinent, with positive MES showing a positive association (*r* = 0.45, BCa 95% CI [0.03, 0.71]) and negative MES showing an inverse association (r = −0.49, BCa 95% CI [−0.79, −0.03]). Bootstrap confidence intervals were consistent with these patterns and did not cross zero, suggesting directionally stable effects within the sample.

**TABLE 2:** Associations Between Mean Edge Strength and Post‐Scan Drinking Outcomes.

Predictors	Outcome	*r*	*p* (raw)	*p* (FDR)	Bootstrap r (BCa 95% CI)
MES Positive	HDD (%)	−0.262	0.279	0.371	−0.262 (−0.660, 0.180)
	Days Abstinent (%)	0.454	0.089	0.178	0.454 (0.032, 0.705)
MES Negative	HDD (%)	0.135	0.582	0.582	0.135 (−0.387, 0.575)
	Days Abstinent (%)	−0.487	0.065	0.178	−0.487 (−0.794, −0.027)

*Note:* Pearson correlation coefficients (r) represent associations between mean edge strength (MES; positive and negative) and drinking outcomes following resting state neuroimaging, including percentage of heavy drinking days (HDD) and percentage of days abstinent. Raw *p*‐values and false discovery rate (FDR) adjusted *p*‐values are reported to account for multiple comparisons. Further, Bias‐corrected and accelerated (BCa) bootstrap estimates are provided with corresponding 95% confidence intervals to assess the stability of effects.

## 4 Discussion

This study provides preliminary evidence that baclofen influences intrinsic functional connectivity in AUD, with dose‐specific effects emerging at high‐dose administration (75 mg/day). We observed dose‐specific connectivity alterations that may reflect neurobiological processes relevant to AUD and baclofen treatment. Although pooled baclofen doses showed no overall effect on intrinsic connectivity, those taking high‐dose baclofen (75 mg/day) evidenced distinct connectivity patterns in brain regions associated with reward processing, stress regulation, and executive control. This was concurrently attributed with lower connectivity within regions implicated in attentional and salience processing, which may suggest a dual mechanism that may be beneficial in AUD. These findings extend previous work showing that baclofen may not only influence cue‐elicited brain activity (Beck et al. 2018; Beraha et al. 2016; Logge et al. 2021), by demonstrating that baclofen may also influence intrinsic functional architecture in neural systems implicated in AUD (Morley et al. 2021). In exploratory analyses, no statistically significant associations were observed between MES and percentage of heavy drinking days or days abstinent; however, moderate effect sizes and bootstrap confidence intervals suggested a positive association for positive MES and an inverse association for negative MES with percentage of days abstinent.

In line with our previous fMRI cue reactivity findings (Logge et al. 2021), high‐dose baclofen compared to placebo was associated with lower intrinsic connectivity with prefrontal regions including the ACC and dlPFC, along with the somatosensory key node, areas known to be highly responsive to substance cues and linked to craving‐related processes (Schacht et al. 2013; Zeng et al. 2021). Consistent with prior work showing altered cue‐modulated connectivity between the VTA, mPFC, and ACC following baclofen treatment (Beck et al. 2018), the lower connectivity observed here within salience and attentional networks may reflect dampened processing of motivationally relevant cues. Lower connectivity in these networks at rest may reflect reduced salience of alcohol‐related cues, potentially supporting greater cognitive control over drinking‐related impulses.

The observed higher connectivity between stress‐ and reward‐related regions (e.g., hypothalamus, amygdala) and the left somatomotor network in the high‐dose baclofen group may be associated with the modulation of stress‐reactive processes linked to alcohol motivation. This aligns with preclinical and clinical evidence suggesting GABAB‐mediated mechanisms can attenuate stress‐related relapse behaviors (Holtyn and Weerts 2022; Logge et al. 2022). Therefore, the higher connectivity with stress and reward regions observed here might reflect an adaptive recalibration of these circuits, which are often dysregulated in AUD (Seo and Sinha 2014; Sinha et al. 2007). Patients with AUD taking high‐dose show lower activity/connectivity in the anterior insula and adjacent OFC compared to low‐dose or placebo‐treated patients (Morley et al. 2021). Chronic alcohol use disrupts the balance between the hypothalamic–pituitary–adrenal (HPA) axis and reward pathways, enhancing stress reactivity and sensitizing individuals to alcohol cues (Stephens and Wand 2012). Therefore, modulation of connectivity in key limbic regions with higher dose baclofen may reduce stress‐associated symptoms in AUD and reduce the likelihood of continued drinking and/or relapse.

Interestingly, these results indicate that high‐dose baclofen exhibits a potential dual mechanism of action. It may enhance connectivity in reward and stress regulation circuits while reducing connectivity in attentional and salience networks. This pattern may foster a more adaptive neural state, reducing automatic responses to alcohol cues and supporting self‐regulation, thereby decreasing relapse vulnerability and promoting recovery. This dual effect could facilitate a decrease in automatic, conditioned responses to alcohol cues (mediated by the salience and attentional networks) while reinforcing the regulation of stress and reward circuits, potentially lowering vulnerability to relapse and craving. These connectivity changes may serve as preliminary biomarkers of baclofen's efficacy, providing early evidence that intrinsic connectivity metrics could help in evaluating pharmacotherapies in AUD.

Although statistically significant associations between functional connectivity and clinical outcomes were not observed within exploratory analyses, this does not preclude the proposed mechanisms and should be interpreted in the context of the study design and sample. In the current research, connectivity metrics were derived using a cross‐sectional design and correlated with aggregated behavioral outcomes over the follow‐up period which may have contributed to limit sensitivity to detect associations between neurobiological treatment effects and behavioral outcomes. However, moderate effect sizes suggested positive MES associations with a larger percentage of abstinence and negative MES an association with a lower percentage of abstinence. Bootstrap confidence intervals were consistent with these patterns, suggesting directionally stable effects within the sample. Together, these findings suggest larger, adequately powered studies are required to examine potential relationships between high‐dose baclofen associated intrinsic functional connectivity and clinical outcomes.

The study contains some notable limitations. The modest sample size limits generalizability of the findings, though in‐line with similar studies evaluating treatment responses in samples with AUD. Moreover, the lack of baseline resting‐state scans precludes conclusions about the direct impact of baclofen on connectivity changes across the treatment period. Additionally, although tobacco use was recorded as part of the clinical assessment, we were limited in the number of covariates that could be included in the statistical models without substantially reducing statistical power. As nicotine use may influence neural responses within reward‐related circuitry, this represents a potential confound that should be considered in future work. In light of these limitations, future work with larger, more diverse samples and longitudinal designs is needed to confirm these preliminary findings and further examine how intrinsic connectivity changes during baclofen treatment relate to clinically meaningful outcomes. Additionally, investigating the interplay between intrinsic connectivity changes and cue reactivity outcomes in a longitudinal context could provide further insights into how baclofen stabilizes neural circuits associated with craving and relapse.

In conclusion, this study reveals that high‐dose baclofen (i.e., at least 75 mg) modulates intrinsic resting‐state functional connectivity in individuals with AUD. Through complex modulation of reward, stress, attentional, and salience networks, baclofen may provide a multifaceted approach reduce drinking behaviors in AUD. These findings support the dual action effect of baclofen, where high‐dose baclofen may be associated with higher connectivity within reward and stress regulation circuits and lower connectivity in attentional and salience networks. This dual mechanism may dampen automatic responses to alcohol cues and strengthen stress regulation, decreasing relapse vulnerability and promoting recovery. These results provide further evidence of baclofen's potential as a dose‐dependent pharmacotherapy for AUD, particularly for patients susceptible to stress‐induced and cue‐elicited relapse. While exploratory analyses did not demonstrate statistically significant associations between mean edge strength (MES) and clinical outcomes, observed trends suggest that these relationships warrant further investigation in larger, adequately powered studies. Furthermore, future research on intrinsic connectivity and cue reactivity together may yield a more comprehensive understanding of the mechanisms through which baclofen and other pharmacotherapies can be used to effectively treat individuals with AUD.

## Funding

This study was supported by a grant from the National Health and Medical Research Council of Australia (P.S.H., A.J.B., K.C.M.).

## Conflicts of Interest

The authors declare no conflicts od interest.

## Supporting information


**Table S1:** Connections with significant seed region Somatomotor 1 from the 256 Schaefer Supplemented with Subcortical Structures parcellation.

## Data Availability

The data that support the findings of this study are available from the corresponding author upon reasonable request.
