# Supplementary material for: Baclofen Modulates Neural Intrinsic Functional Connectivity in Treatment‐Seeking Individuals With Alcohol Use Disorder
Source: Alcohol Clin Exp Res (Hoboken). 2026 Jun 5;50(6):e70332. doi: 10.1111/acer.70332 (PMC13238437; doi:10.1111/acer.70332)
Supplement: Supplementary file 1 — Table S1: Connections with significant seed region Somatomotor 1 from the 256 Schaefer Supplemented with Subcortical Structures parcellation. [file ACER-50-0-s001.docx]

Baclofen modulates neural intrinsic functional connectivity in treatment-seeking individuals with alcohol use disorder

Warren B Logge, PhD ^1,2*^, Paul S Haber PhD ^1,3^, Laurence Mealing ^1,2^, Andrew J Baillie PhD ^4^, Kirsten C Morley PhD ^1,2^

^1^ Edith Collins Centre for Translational Research in Alcohol, Drugs and Toxicology, Royal Prince Alfred Hospital, Sydney Local Health District, Sydney, NSW, Australia

^2^ Specialty of Addiction Medicine, Central Clinical School, Faculty of Medicine and Health, University of Sydney, Sydney, NSW, Australia.

^3^ Drug Health Services, Sydney Local Health District, Sydney, NSW, Australia.

^4^ Faculty of Health Sciences, University of Sydney, SydneyNSW, Australia

**Supplementary Material**

**Methods**

## Image processing

*Anatomical data preprocessing*

A total of 1 T1-weighted (T1w) images were found within the input BIDS dataset.The T1-weighted (T1w) image was corrected for intensity non-uniformity (INU) with N4BiasFieldCorrection (Tustison et al., 2010), distributed with ANTs 2.3.3 (Avants et al., 2008) (RRID:SCR_004757), and used as T1w-reference throughout the workflow. The T1w-reference was then skull-stripped with a Nipype implementation of the antsBrainExtraction.sh workflow (from ANTs), using OASIS30ANTs as target template. Brain tissue segmentation of cerebrospinal fluid (CSF), white-matter (WM) and gray-matter (GM) was performed on the brain-extracted T1w using fast (FSL 5.0.9, RRID:SCR_002823, (Zhang et al., 2001)). Brain surfaces were reconstructed using recon-all (FreeSurfer 6.0.1, RRID:SCR_001847, (Dale et al., 1999)), and the brain mask estimated previously was refined with a custom variation of the method to reconcile ANTs-derived and FreeSurfer-derived segmentations of the cortical gray-matter of Mindboggle (RRID:SCR_002438, (Klein et al., 2017)). Volume-based spatial normalization to two standard spaces (MNI152NLin2009cAsym, MNI152NLin6Asym) was performed through nonlinear registration with antsRegistration (ANTs 2.3.3), using brain-extracted versions of both T1w reference and the T1w template. The following templates were selected for spatial normalization: ICBM 152 Nonlinear Asymmetrical template version 2009c ((Fonov et al., 2009), RRID:SCR_008796; TemplateFlow ID: MNI152NLin2009cAsym), FSL’s MNI ICBM 152 non-linear 6th Generation Asymmetric Average Brain Stereotaxic Registration Model ((Evans et al., 2012), RRID:SCR_002823; TemplateFlow ID: MNI152NLin6Asym).

*Functional data preprocessing*

For each of the 1 BOLD runs found per subject (across all tasks and sessions), the following preprocessing was performed. First, a reference volume and its skull-stripped version were generated using a custom methodology of fMRIPrep. A deformation field to correct for susceptibility distortions was estimated based on fMRIPrep’s fieldmap-less approach. The deformation field is that resulting from co-registering the BOLD reference to the same-subject T1w-reference with its intensity inverted (Huntenburg, 2014; Wang et al., 2017). Registration is performed with antsRegistration (ANTs 2.3.3), and the process regularized by constraining deformation to be nonzero only along the phase-encoding direction, and modulated with an average fieldmap template (Treiber et al., 2016). Based on the estimated susceptibility distortion, a corrected EPI (echo-planar imaging) reference was calculated for a more accurate co-registration with the anatomical reference. The BOLD reference was then co-registered to the T1w reference using bbregister (FreeSurfer) which implements boundary-based registration (Greve and Fischl, 2009). Co-registration was configured with six degrees of freedom. Head-motion parameters with respect to the BOLD reference (transformation matrices, and six corresponding rotation and translation parameters) are estimated before any spatiotemporal filtering using mcflirt (FSL 5.0.9, (Jenkinson et al., 2002)). BOLD runs were slice-time corrected to 1.46s (0.5 of slice acquisition range 0s-2.92s) using 3dTshift from AFNI 20160207 (Cox and Hyde 1997, RRID:SCR_005927). The BOLD time-series were resampled onto the following surfaces (FreeSurfer reconstruction nomenclature): fsaverage. The BOLD time-series (including slice-timing correction when applied) were resampled onto their original, native space by applying a single, composite transform to correct for head-motion and susceptibility distortions. These resampled BOLD time-series will be referred to as preprocessed BOLD in original space, or just preprocessed BOLD. The BOLD time-series were resampled into standard space, generating a preprocessed BOLD run in MNI152NLin2009cAsym space. First, a reference volume and its skull-stripped version were generated using a custom methodology of fMRIPrep. All resamplings can be performed with a single interpolation step by composing all the pertinent transformations (i.e. head-motion transform matrices, susceptibility distortion correction when available, and co-registrations to anatomical and output spaces). Gridded (volumetric) resamplings were performed using antsApplyTransforms (ANTs), configured with Lanczos interpolation to minimize the smoothing effects of other kernels (Lanczos, 1964). Non-gridded (surface) resamplings were performed using mri_vol2surf (FreeSurfer).

*Resting state post-processing of fmriprep outputs*

The eXtensible Connectivity Pipeline (XCP) (Ciric et al., 2017; Satterthwaite et al., 2013) was used to post-process the outputs of fMRIPrep version 20.2.7 (Esteban et al., 2020; Esteban et al., 2019), RRID:SCR_016216). XCP was built with Nipype 1.8.6 ((Gorgolewski et al., 2011), RRID:SCR_002502). Native-space T1w images were transformed to MNI152NLin2009cAsym space at 1 mm3 resolution. F For each of the one BOLD runs found per subject (across all tasks and sessions), the following post-processing was performed. Framewise displacement was calculated using the formula from Power et al. (2014), with a head radius 40.0 mm. Volumes with framewise displacement greater than 0.4 mm were flagged as high-motion outliers for the sake of later censoring (Power et al., 2014). In total, 36 nuisance regressors were selected from the preprocessing confounds, according to the ‘36P’ strategy. These nuisance regressors included six motion parameters, mean global signal, mean white matter signal, mean CSF signal with their temporal derivatives, and the quadratic expansion of six motion parameters, tissues signals and their temporal derivatives (Ciric et al., 2017; Satterthwaite et al., 2013). The BOLD data were despiked with 3dDespike. Nuisance regressors were regressed from the BOLD data using a denoising method based on Nilearn’s approach. Any volumes censored earlier in the workflow were first cubic spline interpolated in the BOLD data. Outlier volumes at the beginning or end of the time series were replaced with the closest low-motion volume’s values, as cubic spline interpolation can produce extreme extrapolations. The timeseries were band-pass filtered using a(n) second-order Butterworth filter, in order to retain signals between 0.01-0.08 Hz. The same filter was applied to the confounds. The resulting time series were then denoised via linear regression, in which the low-motion volumes from the BOLD time series and confounds were used to calculate parameter estimates, and then the interpolated time series were denoised using the low-motion parameter estimates. The interpolated time series were then censored using the temporal mask. The denoised BOLD was smoothed using Nilearn with a Gaussian kernel (FWHM=6.0 mm).

Processed functional timeseries were extracted from the residual BOLD signal with Nilearn’s NiftiLabelsMasker for the Schaefer Supplemented with Subcortical Structures (4S) atlas (Glasser et al., 2013; King et al., 2019; Najdenovska et al., 2018; Pauli et al., 2018; Schaefer et al., 2018) at 256 parcel resolution. Corresponding pair-wise functional connectivity between all regions was computed for each atlas, which was operationalized as the Pearson’s correlation of each parcel’s unsmoothed timeseries. In cases of partial coverage, uncovered voxels (values of all zeros or NaNs) were either ignored, when the parcel had >50.0% coverage, or were set to zero, when the parcel had <50.0% coverage.

Many internal operations of XCP use AFNI (Cox, 1996; Cox and Hyde, 1997), ANTS (Avants et al., 2009), TemplateFlow version 24.2.0 (Ciric et al., 2022), matplotlib version 3.9.2 (Hunter, 2007), Nibabel version 5.2.1 (Brett et al. 2022), Nilearn version 0.10.4 (Abraham et al., 2014), numpy version 2.1.0 (Harris et al., 2020), pybids version 0.17.0 (Yarkoni et al., 2019), and scipy version 1.14.0(Virtanen et al., 2020). For more details, see the xcp_d website <https://xcp-d.readthedocs.io>.

## Supplementary Results

Supplementary Table 1 Connections with significant seed region Somatomotor 1 from the 256 Schaefer Supplemented with Subcortical Structures parcellation

| Seed ROI | Connection |  | Test statistic | p-unc | p-FDR |
| --- | --- | --- | --- | --- | --- |
|  | Side | Connection target |  |  |  |
| Somatomotor 1 | Left |  | F(2,16)=18.88 | .000061 | .015 |
| *Decreased Connectivity* |  |  |  |  |  |
|  | Right | Salience/Ventral Attention Temporal Occ Parietal 3 | T(17)=-4.43 | .000365 | .017 |
|  | Right | Dorsal Attention FEF 1 | T(17)=-3.94 | .00106 | .026 |
|  | Left | Salience/Ventral Attention Med 3 | T(17)=-3.6 | .00219 | .044 |
|  | Right | Dorsal Attention Post 5 | T(17)=-3.44 | .00312 | .044 |
|  | Right | Dorsal Attention Post 4 | T(17)=-3.4 | .00339 | .044 |
|  | Right | Control Parietal 2 | T(17)=-3.39 | .00352 | .044 |
|  | Right | Dorsal Attention PrCv 1 | T(17)=-3.31 | .00417 | .044 |
|  | Right | Control Parietal 1 | T(17)=-3.12 | .00624 | .056 |
|  | Right | Dorsal Attention Post 3 | T(17)=-3.04 | .00735 | .064 |
|  | Left | Control Prefrontal Cortex 3 | T(17)=-2.97 | .00866 | .073 |
|  | Left | Control Parietal 3 | T(17)=-2.93 | .00933 | .073 |
| *Increased Connectivity* |  |  |  |  |  |
|  | Right | Default Temporal 1 | T(17)=5.47 | .000042 | .010 |
|  | Left | Hippocampus | T(17)=4.6 | .000256 | .017 |
|  | Left | Default Prefrontal Cortex 2 | T(17)=4.45 | .000351 | .017 |
|  | Right | Control Prefrontal Cortex 1 | T(17)=4.39 | .000397 | .017 |
|  | Left | Hypothalamus (HTH) | T(17)=4.37 | .000414 | .017 |
|  | Left | Default Temporal 3 | T(17)=4.27 | .000516 | .018 |
|  | Left | Mamilliary Nucleus (MN) | T(17)=4.03 | .000872 | .026 |
|  | Left | Control Orbitofrontal Cortex 1 | T(17)=3.96 | .00101 | .026 |
|  | Left | Default Parahippocampus (PHC) 1 | T(17)=3.86 | .00125 | .028 |
|  | Right | Hypothalamus (HTH) | T(17)=3.57 | .00238 | .044 |
|  | Right | Limbic Orbitofrontal Cortex 3 | T(17)=3.47 | .0029 | .044 |
|  | Right | Amygdala | T(17)=3.47 | .00291 | .044 |
|  | Left | Default Temporal 1 | T(17)=3.42 | .00326 | .044 |
|  | Left | Substantia nigra pars compacta/parabrachial pigmented nucleus/Ventral Tegmental Area (SNc PBP VTA) | T(17)=3.35 | .00381 | .044 |
|  | Left | Limbic Orbitofrontal Cortex 1 | T(17)=3.3 | .00422 | .044 |
|  | Right | Limbic Orbitofrontal Cortex 2 | T(17)=3.29 | .00431 | .044 |
|  | Right | Default Prefrontal Cortex/Dorsal;Medial 1 | T(17)=3.29 | .00435 | .044 |
|  | Left | Default Prefrontal Cortex 1 | T(17)=3.23 | .00489 | .047 |
|  | Left | Default Prefrontal Cortex 4 | T(17)=3.13 | .00609 | .056 |
|  | Right | Subthalamic Nucleus (STH) | T(17)=2.93 | .00928 | .073 |

## References

Abraham, A, Pedregosa, F, Eickenberg, M *et al.* (2014) Machine learning for neuroimaging with scikit-learn. *Front Neuroinform* **8**.

Avants, BB, Epstein, CL, Grossman, M, Gee, JC (2008) Symmetric diffeomorphic image registration with cross-correlation: Evaluating automated labeling of elderly and neurodegenerative brain. *Med Image Anal* **12**: 26-41.

Avants, BB, Tustison, N, Song, G (2009) Advanced normalization tools (ANTS). *Insight j* **2**: 1-35.

Ciric, R, Thomas, AW, Esteban, O, Poldrack, RA (2022) Differentiable programming for functional connectomics. *arXiv preprint arXiv:220600649*.

Ciric, R, Wolf, DH, Power, JD *et al.* (2017) Benchmarking of participant-level confound regression strategies for the control of motion artifact in studies of functional connectivity. *Neuroimage* **154**: 174-87.

Cox, RW (1996) AFNI: Software for Analysis and Visualization of Functional Magnetic Resonance Neuroimages. *Comput Biomed Res* **29**: 162-73.

Cox, RW and Hyde, JS (1997) Software tools for analysis and visualization of fMRI data. *NMR Biomed* **10**: 171-78.

Dale, AM, Fischl, B, Sereno, MI (1999) Cortical Surface-Based Analysis: I. Segmentation and Surface Reconstruction. *Neuroimage* **9**: 179-94.

Esteban, O, Ciric, R, Finc, K *et al.* (2020) Analysis of task-based functional MRI data preprocessed with fMRIPrep. *Nat Protoc* **15**: 2186-202.

Esteban, O, Markiewicz, CJ, Blair, RW *et al.* (2019) fMRIPrep: a robust preprocessing pipeline for functional MRI. *Nature Methods* **16**: 111-16.

Evans, AC, Janke, AL, Collins, DL, Baillet, S (2012) Brain templates and atlases. *Neuroimage* **62**: 911-22.

Fonov, VS, Evans, AC, McKinstry, RC, Almli, CR, Collins, DL (2009) Unbiased nonlinear average age-appropriate brain templates from birth to adulthood. *Neuroimage* **47**: S102.

Glasser, MF, Sotiropoulos, SN, Wilson, JA *et al.* (2013) The minimal preprocessing pipelines for the Human Connectome Project. *Neuroimage* **80**: 105-24.

Gorgolewski, K, Burns, CD, Madison, C *et al.* (2011) Nipype: a flexible, lightweight and extensible neuroimaging data processing framework in python, *Front Neuroinform*, Vol. 5, pp. 13.

Greve, DN and Fischl, B (2009) Accurate and robust brain image alignment using boundary-based registration. *Neuroimage* **48**: 63-72.

Harris, CR, Millman, KJ, van der Walt, SJ *et al.* (2020) Array programming with NumPy. *Nature* **585**: 357-62.

Huntenburg, JM (2014) Evaluating nonlinear coregistration of BOLD EPI and T1w images: Freie Universität Berlin.

Hunter, JD (2007) Matplotlib: A 2D Graphics Environment. *Computing in Science &amp; Engineering* **9**: 90-95.

Jenkinson, M, Bannister, P, Brady, M, Smith, S (2002) Improved Optimization for the Robust and Accurate Linear Registration and Motion Correction of Brain Images. *Neuroimage* **17**: 825-41.

King, M, Hernandez-Castillo, CR, Poldrack, RA, Ivry, RB, Diedrichsen, J (2019) Functional boundaries in the human cerebellum revealed by a multi-domain task battery. *Nat Neurosci* **22**: 1371-78.

Klein, A, Ghosh, SS, Bao, FS *et al.* (2017) Mindboggling morphometry of human brains. *PLoS Comput Biol* **13**: e1005350.

Lanczos, C (1964) Evaluation of Noisy Data. *Journal of the Society for Industrial and Applied Mathematics Series B Numerical Analysis* **1**: 76-85.

Najdenovska, E, Alemán-Gómez, Y, Battistella, G *et al.* (2018) In-vivo probabilistic atlas of human thalamic nuclei based on diffusion- weighted magnetic resonance imaging. *Scientific Data* **5**: 180270.

Pauli, WM, Nili, AN, Tyszka, JM (2018) A high-resolution probabilistic in vivo atlas of human subcortical brain nuclei. *Scientific Data* **5**: 180063.

Power, JD, Mitra, A, Laumann, TO, Snyder, AZ, Schlaggar, BL, Petersen, SE (2014) Methods to detect, characterize, and remove motion artifact in resting state fMRI. *Neuroimage* **84**: 320-41.

Satterthwaite, TD, Elliott, MA, Gerraty, RT *et al.* (2013) An improved framework for confound regression and filtering for control of motion artifact in the preprocessing of resting-state functional connectivity data. *Neuroimage* **64**: 240-56.

Schaefer, A, Kong, R, Gordon, EM *et al.* (2018) Local-Global Parcellation of the Human Cerebral Cortex from Intrinsic Functional Connectivity MRI. *Cerebral cortex (New York, NY : 1991)* **28**: 3095-114.

Treiber, JM, White, NS, Steed, TC *et al.* (2016) Characterization and Correction of Geometric Distortions in 814 Diffusion Weighted Images. *PLoS One* **11**: e0152472.

Tustison, NJ, Avants, BB, Cook, PA *et al.* (2010) N4ITK: Improved N3 Bias Correction. *IEEE Trans Med Imaging* **29**: 1310-20.

Virtanen, P and Gommers, R and Oliphant, TE *et al.* (2020) SciPy 1.0: fundamental algorithms for scientific computing in Python. *Nature Methods* **17**: 261-72.

Wang, S, Peterson, DJ, Gatenby, JC, Li, W, Grabowski, TJ, Madhyastha, TM (2017) Evaluation of Field Map and Nonlinear Registration Methods for Correction of Susceptibility Artifacts in Diffusion MRI, *Front Neuroinform*, Vol. 11, pp. 17.

Yarkoni, T, Markiewicz, CJ, de la Vega, A *et al.* (2019) PyBIDS: Python tools for BIDS datasets. *J Open Source Softw* **4**.

Zhang, Y, Brady, M, Smith, S (2001) Segmentation of brain MR images through a hidden Markov random field model and the expectation-maximization algorithm. *IEEE Trans Med Imaging* **20**: 45-57.
